# Sepsityper^®^ Kit versus In-House Method in Rapid Identification of Bacteria from Positive Blood Cultures by MALDI-TOF Mass Spectrometry

**DOI:** 10.3390/life12111744

**Published:** 2022-10-30

**Authors:** Gabrijela Perše, Ivana Samošćanec, Zrinka Bošnjak, Ana Budimir, Tomislav Kuliš, Ivana Mareković

**Affiliations:** 1 Department of Clinical and Molecular Microbiology, University Hospital Centre Zagreb, 10 000 Zagreb, Croatia; 2 Department of Clinical Microbiology and Hospital Infections, University Hospital “Sveti Duh”, 10 000 Zagreb, Croatia; 3 School of Medicine, University of Zagreb, 10 000 Zagreb, Croatia; 4 Department of Urology, University Hospital Centre Zagreb, 10 000 Zagreb, Croatia

**Keywords:** MALDI-TOF MS, blood culture, identification, in-house method, Sepsityper^®^ Kit

## Abstract

In order to further accelerate pathogen identification from positive blood cultures (BC), various sample preparation protocols to identify bacteria with MALDI-TOF MS directly from positive BCs have been developed. We evaluated an in-house method in comparison to the Sepsityper^®^ Kit (Bruker Daltonics, Bremen, Germany) as well as the benefit of an on-plate formic acid extraction step following positive signal by the BACTEC^TM^ FX system. Confirmation of identification was achieved using subcultured growing biomass used for MALDI-TOF MS analysis. A total of 113 monomicrobial positive BCs were analyzed. The rates of Gram-positive bacteria correctly identified to the genus level using in-house method and Sepsityper^®^ Kit were 63.3% (38/60) and 81.7% (49/60), respectively (*p* = 0.025). Identification rates at species level for Gram-positive bacteria with in-house method and Sepsityper^®^ kit were 30.0% (18/60) and 66.7% (40/60), respectively (*p* < 0.001). Identification rates of Gram-negative bacteria were similar with the in-house method and Sepsityper^®^ Kit. Additional on-plate formic acid extraction demonstrated significant improvement in the identification rate of Gram-positive bacteria at both genus and species level for both in-house (*p* = 0.001, *p* < 0.001) and Sepsityper^®^ Kit methods (*p* = 0.007, *p* < 0.001). Our in-house method is a candidate for laboratory routines with Sepsityper^®^ Kit as a back-up solution when identification of Gram-positive bacteria is unsuccessful.

## 1. Introduction

Bloodstream infections (BSI) are the leading cause of morbidity and mortality worldwide and as a life-threatening condition require prompt diagnosis and treatment. In Europe and North America BSI incidence ranges between 113 and 204 per 100,000 people [1,2,3,4]. Furthermore, the incidence of BSI is rising, probably related to an aging population and an increasing prevalence of underlying conditions and invasive procedures [5]. Mortality estimates vary with country from 15.3% to 40.0% [6]. Rapid and accurate identification of the causative pathogen and timely administration of targeted antimicrobial treatment is crucial for reducing mortality in septic patients as well as the ongoing spread of antimicrobial resistance [7,8].

The gold standard for etiological diagnosis of BSI is still blood culture (BC). The classical approach to BC processing used until 2010 was slow and took >24 h due to the subsequent overnight subculture onto solid media for final identification by phenotypical and biochemical analysis [9].

After 2010 matrix-assisted laser desorption ionization time-of-flight mass spectrometry (MALDI-TOF MS) with extensive coverage, low cost and user-friendliness revolutionized the field of clinical microbiology, enabling the identification of bacteria from grown colonies within minutes. The use of MALDI-TOF MS in BC processing was further advanced by applying it sooner, namely on a thin layer of microorganisms grown on the surface of the plate after short incubation allowed for very early and reliable identification. According to the published literature, the mean incubation time needed to achieve species level identification is 5.9 and 2.0 h for Gram-positive aerobic cocci and Gram-negative rods, respectively [10,11].

In order to further accelerate pathogen identification from positive BCs, various sample preparation protocols to identify bacteria with MALDI-TOF MS directly from positive BCs have been developed. The purpose of these protocols is to remove blood cells and host proteins from the positive BC broth prior to MALDI-TOF MS analysis of the species-specific protein spectra of the bacteria to be revealed. Nowadays, there are different in-house methods, as well as commercially available kits, for positive BC broth sample preparation to make it suitable for MALDI-TOF MS analysis. The in-house developed protocols are based on the obtainment of purified microorganism cells using either various lysis reagents, such as saponin, sodium dodecyl sulfate (SDS), and ammonium chloride, or stepwise centrifugation in order to separate blood cell components from bacterial cells [12,13,14,15,16,17,18,19,20]. There are also three commercial kits currently in use: Sepsityper^®^ Kit (Bruker Daltonics GmbH, Bremen, Germany), Vitek MS blood culture kit (bioMérieux, Marcy-l’Étoile, France) and rapid BACpro^®^ II (Nittobo Medical Co., Tokyo, Japan) [12,13,20]. Among the three commercially available kits, the Sepsityper^®^Kit is the most widely used CE-IVD labeled and FDA approved commercial kit and a meta-analysis of its performance has indicated that this method provided reliable species-level identification for approximately 80% of 3320 positive blood culture cases tested [21].

An additional challenge when applying MALDI-TOF MS directly on positive BCs is analysis of the results, with scores usually lower in comparison to those obtained from colonies grown on solid agar. Sepsityper^®^ module was recently developed for improved MALDI-TOF MS identification directly from BCs. Sepsityper^®^ module analyzes mass spectra with two main differences in comparison to standard analysis. Firstly, the lower mass range threshold used for peak picking is at 4 kDa instead of 3 kDa as for standard samples. This is due to the presence of multiple peaks that originate from blood cells between 3 and 4 kDa. Raising the peak picking threshold means that these peaks are ignored during processing. Secondly, confidence thresholds when interpreting scores are lower in comparison to standard samples [13].

We adapted an in-house method from previously described protocols and evaluated it in comparison to the Sepsityper^®^ Kit for identification of bacteria directly from positive BC bottles [13,14,18,20]. Additionally, we investigated the benefit of using an on-plate formic acid extraction step. As our in-house method is equal in terms the hands-on time required, but is inexpensive, our goal was to determine whether it would be preferable for implementation in a laboratory routine.

## 2. Materials and Methods

The study was performed between February 2022 and July 2022 at the Department of Clinical and Molecular Microbiology, University Hospital Centre Zagreb, Zagreb, Croatia. UHCZ is a 1510-bed tertiary care hospital with 131 ICU beds. The total number of BC bottles processed each year is ca. 25,000. The BC were collected at the clinical wards and then transferred to the laboratory. Three different BC bottles, i.e., BD BACTEC Plus Aerobic/F, BD BACTEC Plus Anaerobic/F and BD BACTEC PEDS Plus/F were used. The BC bottles were incubated in BACTEC^TM^ FX BC systems (Becton Dickinson, Heidelberg, Germany) until positive signal or for 5 days if negative. Following the detection of microbial growth, Gram stain was performed. Following the Gram stain, only monomicrobial BCs were included in the study. In addition to the routinely used conventional method, each positive BC bottle broth was processed simultaneously by the in-house method and Sepsityper^®^ Kit before MALDI-TOF MS analysis. In contrast to the in-house method, Sepsityper^®^ Kit protocol contains a lysis buffer which collects bacteria by lysing blood cell components.

In the routinely used conventional method positive BC bottles were subcultured on solid media and overnight growth at 37 °C in 5% CO_2_ was used for MALDI-TOF MS identification (MALDI Biotyper Microflex LT/SH, Bruker Daltonics GmbH, Bremen, Germany). The conventional method was used as a reference method for confirmation of identification.

In the in-house method a volume of 5 mL of BC broth was transferred from each positive bottle into VACUETTE^TM^ Z Serum Sep Clot Activator Tube (Greiner Bio-One, Monroe, NC, USA) and centrifuged at 3000 rounds per minute (rpm) for 10 min at room temperature. The supernatant was cautiously discarded. A formed pellet at the surface of the gel was then resuspended into 1 mL of demineralized water. Samples were vortexed prior to final centrifugation at 14,000 rpm for 2 min. Once again, supernatant was discarded and remaining the pellet was deposited onto MALDI-target plate.

**Figure 1:**
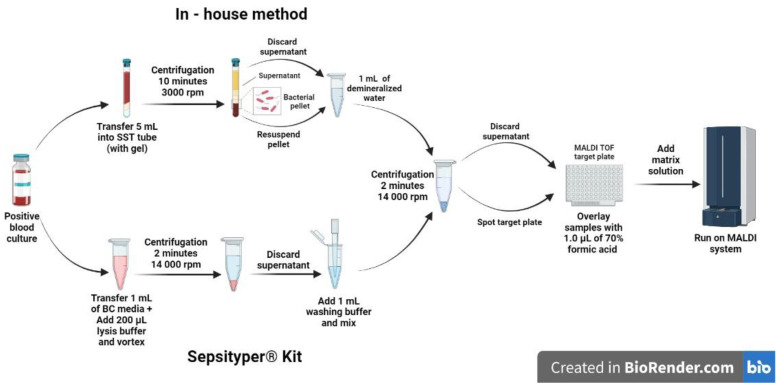
Overview of the two pretreatment protocols that were used for rapid identification frompositive blood cultures (in-house method in the upper part and Sepsityper^®^ Kit in the lower part of the figure).

When Sepsityper^®^ Kit was used, according to manufacturer instructions 1 mL of positive BC broth was transferred to Eppendorf tube and 200 µL of lysis buffer was added. The sample was thoroughly vortexed prior to 2-min centrifugation at 14.000 rpm. Supernatant was carefully removed and remaining pellet resuspended in 1 mL of washing buffer. Following second centrifugation (2 min at 14,000 rpm), supernatant was discarded and pellet was deposited onto MALDI-target plate. In Figure 1 in-house and Sepsityper^®^ Kit protocols used in the study are summarized.

For MALDI-TOF MS analysis for both the in-house method and the Sepsityper^®^ Kit, two spots with and two spots without a formic acid extraction step on target plate were included (eight spots in total for each positive BC). Namely, when the bacterial pellet on the target plate was dry, two out of four spots obtained with both in-house and Sepsityper^®^ Kit were overlaid with 1 µL of 70% of formic acid and air dried. Next, the same volume of alpha-cyano-4-hydroxycinnamic acid (HCCA) matrix solution was placed on each spot. The Bacterial Test Standard (Bruker Daltonics GmbH, Bremen, Germany) was used for calibration purposes. Identification scores obtained by MALDI-TOF MS were interpreted according to the manufacturer’s recommendations. The higher score of two was used for the final analysis. For the in-house method standard cut-off values were used as follows: score value ≥ 2.000 indicated correct identification to the species level, score value 1.700–1.999 indicated correct identification to the genus level and score value < 1.700 indicated no reliable identification. For the Sepsityper^®^ kit, the Sepsityper^®^ software module was used with lower cut-off values as follows: score value ≥ 1.800 indicated correct identification to the species level, score value 1.600–1.799 indicated correct identification to the genus level and score value < 1.600 indicated no reliable identification.

Categorical variables are reported as count and percentages. To check for the difference between categorical variables we have used Chi-square test or Fischer’s exact test as applicable. For the statistical analyses we have used MedCalc Statistical Software version 20.0.4 (MedCalc Software Ltd., Ostend, Belgium). All reported *p* values are two-sided and statistical significance was defined as *p* < 0.05.

## 3. Results

During the study period a total of 113 monomicrobial positive BCs were collected and identified by routine microbiological procedures. In total 60 (53.1%) Gram-positive and 53 (46.9%) Gram-negative bacterial isolates were detected. The results of identification by MALDI-TOF MS analysis of positive BCs using the in-house method and Sepsityper^®^ Kit are shown in Table 1.

The overall MALDI-TOF MS-based identification rates at genus and species level for samples prepared using the in-house method were 78.8% (89/113) and 54.9% (62/113), respectively. For the Sepsityper^®^ Kit, 87.6% (99/113) and 77.0% (87/113) of microorganisms were correctly identified to the genus and species level, respectively. There was no significant difference between the in-house method and Sepsityper^®^ Kit for MALDI-TOF-based identification directly from positive blood cultures at the genus but the difference was significant at the species level (*p* = 0.076, *p* = 0.001) (Table 2).

The rates of Gram-positive bacteria correctly identified to the genus level using the in-house method and Sepsityper^®^ Kit were 63.3% (38/60) and 81.7% (49/60), respectively. There was significant difference between the in-house method and Sepsityper^®^ Kit for the identification of Gram-positive bacteria at genus level (*p* = 0.025). Identification rates on species level for Gram-positive bacteria with the in-house method and the Sepsityper^®^ kit were 30.0% (18/60) and 66.7% (40/60), respectively. There was significant difference between the in-house method and Sepsityper^®^ kit for the identification of Gram-positive bacteria at species level (*p* < 0.001) (Table 2). Identification rates of Gram-negative bacteria were similar with the in-house method and Sepsityper^®^ Kit for both genus (96.2% (51/53) versus 94.3% (50/53), *p* = 1.000) and species level (83.0% (44/53) versus 88.7% (47/53), *p* = 0.405) (Table 2).

Misidentified Gram-positive microorganisms included *Micrococcus luteus* (*n* = 1) and *Parvimonas micra* (*n* = 1). In case of *Micrococcus luteus*, the in-house method yielded no possible identification, with a MALDI-TOF MS score < 1.7, whereas the Sepsityper^®^ Kit misidentified this isolate as *Lactobacillus helveticus* (score 1.79). *Parvimonas micra* was misidentified by the in-house method and the Sepsityper^®^ Kit as *Lancefieldella parvula* with scores of 1.95 and 1.68, respectively.

Another aim of the study was to analyze the impact of additional on-plate formic acid extraction on MALDI Biotyper identification rates.

In-house method with additional on-plate formic acid extraction showed a significantly higher overall identification rate in comparison to the in-house method without that step for both genus (77.0% (87/113) vs. 46.0% (52/113), *p* < 0.001) and species level (52.2% (59/113) vs. 34.5% (39/113), *p* = 0.007). Stratification according to Gram stain results demonstrated significant improvement in identification rate of Gram-positive bacteria on both genus (8.3% (5/60) vs. 63.3% (38/60), *p* ≤ 0.001) and species level (5.0% (3/60) vs. 30.0% (18/60), *p* = 0.001). It showed no significant improvement for identification of Gram-negative bacteria for either genus (88.7% (47/53) vs. 92.5% (49/53), *p* = 0.742) or species level (67.9% (36/53) vs. 77.4% (41/53, *p* = 0.278) (Table 3).

The Sepsityper^®^ Kit method with additional on-plate formic acid extraction showed a significantly higher overall identification rate in comparison to the Sepsityper^®^ Kit method without that step for both genus (84.1% (95/113) vs. 69.9% (79/113), *p* = 0.012) and species level (72.6% (82/113) vs. 55.8% (63/113), *p* = 0.009). Stratification according to Gram stain results demonstrated significant improvement in the identification rate of Gram-positive bacteria on both genus (from 55.0% (33/60) to 78.3% (47/60), *p* = 0.007) and species level (from 31.7% (19/60) to 65.0% (39/60), *p* < 0.001). There was no statistically significant improvement for identification of Gram-negative bacteria on either genus (from 86.8% (46/53) to 90.6% (48/53), *p* = 0.761) or species level (from 83.0% (44/53) to 81.1% (43/53), *p* = 0.801) (Table 4).

## 4. Discussion

Identification of bacteria directly from positive BC broth by MALDI-TOF MS is an important step forward in diagnostics of BSIs. One of the main advantages is the potential to identify over 2200 different bacterial species with profiles present in the database. In comparison, commercial platforms based on multiplex PCR or microarray technology also allow identification of bacterial pathogens directly from positive BC within 1–4 h. However, their main drawbacks are limited panels of pathogens, the possibility of processing a few samples simultaneously and a significantly higher cost [9,22,23,24].

The problem with MALDI-TOF MS identification directly from positive BC broths is the necessity of prior sample processing in order to remove other disturbing signals originating from human cells, culture media and other debris present in the BC broth [13]. Commercial kits for sample processing are creating additional costs for laboratory budgets. Therefore, different in-house methods are described in the literature using different low-cost solutions prepared in the laboratory (for example saponin, etc.) [12,14,20]. Additionally, in-house methods have also been described with additional expenses including only centrifugation tubes and demineralized water used for the washing step. The methods with serum separator tubes overcome the prolonged turnaround time when the protocol includes repetitive washing and centrifugation steps [18]. Therefore, in our study we investigated one such method that we adapted from previously described protocols [13,14,15,16,18,20].

In our study, performed on 113 monomicrobial positive BC broths, there was significant difference between the in-house (54.9%) method and Sepsityper^®^ Kit (77.0%) for MALDI-TOF-based overall identification rate directly from positive blood cultures at the species level. Identification rates of Gram-negative bacteria were similar to the in-house method (96.2%, 83.0%) and Sepsityper^®^ Kit (94.3%, 88.7%) for both genus and species level, but there was significant difference between the in-house method (63.3%, 30.0%) and Sepsityper^®^ Kit (81.7%, 66.7%) for the identification of Gram-positive bacteria at genus and species level. Identification rates of the Sepsityperkit^®^ Kit obtained in our study were similar to those reported in a meta-analysis—90% on average for Gram-negative and 60% on average for Gram-positive bacteria [21]. Both the in-house method and Sepsityper^®^ Kit were more successful in identification of Gram-negative bacteria by MALDI-TOF MS directly from BCs. Similar findings have been shown in previous studies and the possible explanations include the thick cell wall in Gram-positive bacteria making protein extraction more demanding, adherence of Gram-positive bacteria to erythrocytes and their removal with the serum or smaller pellet due to slower growth Gram-positive bacteria in positive BCs [13,15,16,18,21]. Still, the Sepsityper^®^ Kit was more successful in identification of Gram-positive bacteria, which can be explained with the use of a lysis buffer and Sepsityper^®^ module with lower cut-off thresholds in analysis of results that were not included in the in-house method. In other previously published studies, the use of the Sepsityper module^®^ also significantly improved the overall identification rates of bacteria, especially of Gram-positive bacteria [13,25].

In our study additional on-plate formic acid extraction demonstrated significant improvement in the identification rate of Gram-positive but not Gram-negative bacteria on both genus and species level for both the in-house and the Sepsityper^®^ Kit method. Originally described, a simple protein extraction method with formic acid performed directly on target plate was shown to be at least as good as standard extraction for the identification of staphylococci [26]. Its effect on the identification rate of Gram-positive bacteria directly from positive BCs is demonstrated elsewhere [25,27,28]

Only 2/113 samples were misidentified. Identification of *M. luteus* as *L. helveticus* would have been ignored on the basis of a Gram stain result that showed Gram-positive cocci. A possible explanation could be poor cleaning and the presence of residual materials of the reusable target plate. In our laboratory we used trichlorofluoroacteate (TFA) protocol as suggested by manufacturer. Other protocols, for example with ethanol and mechanical cleaning of target plates, have been shown to be insufficient to properly clean MALDI-TOF microplates [29]. False identification of *P. micra* as *L. parvula* would not have led to inadequate treatment because both species belong to the group of Gram-positive anaerobic cocci. Although recently it has been demonstrated that only 2.1% of the anaerobic isolates could not be reliably identified by the MALDI-TOF MS method when biomass from colonies is used, a few studies with a small number of samples have referred to MALDI-TOF performance in identification of anaerobes directly from positive BCs [12,13,30].

There are limitations in the present study. Firstly, the results of direct MALDI-TOF MS identification were not used for adjustment of antimicrobial treatment in patients whose BCs were included in the study. However, previous studies have shown that identification by MALDI-TOF MS directly from positive BCs may improve treatment quality and patient outcomes only in combination with an antimicrobial stewardship program [31,32,33]. Secondly, we did not compare identification rates among three different BC bottle types used. Previous studies have demonstrated a significantly higher identification rate when BD BACTEC anaerobic bottles were used. The reasons for that could include the way in which resin beads in aerobic BD BACTEC bottles interfere with protein extraction or how saponin in BD BACTEC anaerobic bottles enhances red blood cell lysis. Furthermore, studies using the BACTEC system report higher identification rates compared to those using BacT/Alert bottles [21,25].

In conclusion, we adapted an easy, fast and inexpensive protocol for the identification of bacteria from positive BCs with identification rate of Gram-negative bacteria comparable to commercial Sepsityper^®^ kit. However, extraction with Sepsityper^®^ Kit is the optimal solution in providing a greater rate of precision for the identification of Gram-positive bacteria with additional on-plate extraction with formic acid showing benefit in identification rate. Our in-house method is a candidate for laboratory routine with Sepsityper^®^ Kit as a back-up solution when identification of Gram-positive bacteria is unsuccessful.

## Figures and Tables

**Table 1:** MALDI Biotyper scores for the identification of bacteria from positive monomicrobial blood cultures using the in-house method and the Sepsityper^®^ Kit.

Microorganisms as Identified by the Routine Method	N	In-House Method			Sepsityper Kit		
		<1.700	1.700–1.999	≥2.000	<1.600	1.600–1.799	≥1.800
**Gram-negative bacteria**	**53**	**2**	**7**	**44**	**3**	**3**	**47**
*Acinetobacter baumannii*	7		1	6	1	1	5
*Bacteroides fragilis*	1			1	1		
*Enterobacter asburiae*	1			1			1
*Enterobacter cloacae*	2			2			2
*Escherichia coli*	10		2	8		1	9
*Klebsiella aerogenes*	1		1				1
*Klebsiella oxytoca*	1			1			1
*Klebsiella pneumoniae*	12	1	1	10	1	1	10
*Providentia stuartii*	1			1			1
*Pseudomonas aeruginosa*	8			8			8
*Pseudomonas stutzeri*	1			1			1
*Salmonella sp*	1			1			1
*Serratia marcescens*	5		1	4			5
*Stenotrophomonas maltophilia*	2	1	1				2
**Gram-positive bacteria**	**60**	**22**	**20**	**18**	**11**	**9**	**40**
*Corynebacterium striatum*	1	1			1		
*Dietzia papillomatosis*	1	1				1	
*Enterococcus faecalis*	2		1	1			2
*Enterococcus faecium*	6		1	5			6
*Micrococcus luteus* ^1^	1						
*Parvimonas micra* ^1^	1						
*Staphylococcus aureus*	9	5	2	2	2		7
*Staphylococcus capitis*	2		1	1			2
*Staphylococcus cohnii*	1	1					1
*Staphylococcus epidermidis*	18	8	8	2	4	4	10
*Staphylococcus haemolyticus*	12	3	6	3	1	4	7
*Staphylococcus hominis*	3			3			3
*Staphylococcus lungdunensis*	1			1			1
*Streptococcus anginosus*	1		1				1
*Streptococcus gordonii*	1	1			1		
**Total**	**113**	**24**	**27**	**62**	**14**	**12**	**87**

^1^ misidentified isolates.

**Table 2:** Comparison of two preparation methods for MALDI-TOF MS-based identification of microorganisms from positive blood cultures.

Microorganism Group	In-House Method		Sepsityper^®^ Kit		In-House Method Versus Sepsityper^®^ Kit	
	Identified to the Genus Level (Score ≥ 1.700, %)	Identified to the Species Level (Score ≥ 2.000, %)	Identified to the Genus Level (Score ≥ 1.600, %)	Identified to the Species Level (Score ≥ 1.800, %)	*p*-Value (Genus Level Identification)	*p*-Value (Species Level Identification)
Gram-negative bacteria	96.2	83.0	94.3	88.7	1.000	0.405
Gram-positive bacteria	63.3	30.0	81.7	66.7	0.025	<0.001
Total	78.8	54.9	87.6	77.0	0.076	0.001

**Table 3:** Impact of additional on-plate formic acid extraction on MALDI Biotyper scores for microorganisms’ identification using an in-house method.

Microorganism Group	Without Additional On-Plate Formic Acid N (%)		With Additional On-Plate Formic Acid N (%)		In-House Method without Versus with Formic Acid	
	Genus Level ≥ 1.700	Species Level ≥ 2.000	Genus Level ≥ 1.700	Species Level ≥ 2.000	*p*-Value (Genus Identification	*p*-Value (Species Identification)
Gram-negative bacteria (*n* = 53)	47 (88.7)	36 (67.9)	49 (92.5)	41 (77.4)	0.742	0.278
Gram-positive bacteria (*n* = 60)	5 (8.3)	3 (5.0)	38 (63.3)	18 (30.0)	<0.001	0.001
Total (*n* = 113)	52 (46.0)	39 (34.5)	87 (77.0)	59 (52.2)	<0.001	0.007

**Table 4:** Impact of additional on-plate formic acid extraction on MALDI Biotyper scores for microorganisms’ identification using Sepsityper^®^ Kit.

Microorganism Group	Without AdditionalOn-Plate Formic Acid N (%)		With Additional On-Plate Formic Acid N (%)		Sepsityper^®^ Kit without Versus with Formic Acid	
	Genus Level ≥ 1.600	Species Level ≥ 1.800	Genus Level ≥ 1.600	Species Level ≥ 1.800	*p*-Value (Genus Identification	*p*-Value (Species Identification)
Gram-negative bacteria (*n* = 53)	46 (86.8)	44 (83.0)	48 (90.6)	43 (81.1)	0.761	0.801
Gram-positive bacteria (*n* = 60)	33 (55.0)	19 (31.7)	47 (78.3)	39 (65.0)	0.007	<0.001
Total (*n* = 113)	79 (69.9)	63 (55.8)	95 (84.1)	82 (72.6)	0.012	0.009
